# Transcriptome Analysis of Diurnal and Nocturnal-Warmed Plants, the Molecular Mechanism Underlying Cold Deacclimation Response in *Deschampsia antarctica*

**DOI:** 10.3390/ijms241311211

**Published:** 2023-07-07

**Authors:** Dariel López, Giovanni Larama, Patricia L. Sáez, León A. Bravo

**Affiliations:** 1 Departamento de Ciencias Agronómicas y Recursos Naturales, Facultad de Ciencias Agropecuarias y Medioambiente and Center of Plant, Soil Interactions and Natural Resources Biotechnology, Scientific and Technological Bioresource Nucleus, Universidad de La Frontera, Temuco 4811230, Chile; d.lopez07@ufromail.cl (D.L.); patrisaezd@gmail.com (P.L.S.); 2 Biocontrol Research Laboratory and Scientific and Technological Bioresource Nucleus, Universidad de La Frontera, Temuco 4811230, Chile; giovanni.larama@ufrontera.cl

**Keywords:** Antarctic plant, asymmetric warming, CBF, climate change, cold deacclimation, gene expression, RNA-seq

## Abstract

Warming in the Antarctic Peninsula is one of the fastest on earth, and is predicted to become more asymmetric in the near future. Warming has already favored the growth and reproduction of Antarctic plant species, leading to a decrease in their freezing tolerance (deacclimation). Evidence regarding the effects of diurnal and nocturnal warming on freezing tolerance-related gene expression in *D. antarctica* is negligible. We hypothesized that freezing tolerance-related gene (such as CBF-regulon) expression is reduced mainly by nocturnal warming rather than diurnal temperature changes in *D. antarctica.* The present work aimed to determine the effects of diurnal and nocturnal warming on cold deacclimation and its associated gene expression in *D. antarctica*, under laboratory conditions. Fully cold-acclimated plants (8 °C/0 °C), with 16h/8h thermoperiod and photoperiod duration, were assigned to four treatments for 14 days: one control (8 °C/0 °C) and three with different warming conditions (diurnal (14 °C/0 °C), nocturnal (8 °C/6 °C), and diurnal-nocturnal (14 °C/6 °C). RNA-seq was performed and differential gene expression was analyzed. Nocturnal warming significantly down-regulated the CBF transcription factors expression and associated cold stress response genes and up-regulated photosynthetic and growth promotion genes. Consequently, nocturnal warming has a greater effect than diurnal warming on the cold deacclimation process in *D. antarctica*. The eco-physiological implications are discussed.

## 1. Introduction

The phenomenon known as climate change, implying shifts in temperature, precipitation, and CO_2_ levels among other factors, represents a major threat to agricultural and natural plant populations. Deforestation and fossil fuel burning have increased the concentration of greenhouse gases in the atmosphere, contributing to increases in the global average surface temperature of 1.0–3.7 °C during the past century [1,2]. It has been estimated that global warming will increase the frequency and intensity of stochastic extreme temperature events (freezing or heat waves), with changes in precipitation patterns during this century [2]. In addition, an asymmetric warming trend is expected, with a greater increase in minimum temperatures (nocturnal) with respect to maximum temperatures (diurnal) [2,3]. Furthermore, the warming process has been amplified in polar and high-mountain regions [4].

The climate of the Antarctic Peninsula and its associated islands has shown increases in the average annual temperature ca. 3.7 °C century^−1^ [5]. With longer and warmer growing seasons for the plant species that inhabit the Antarctic Peninsula, reproductive capacity and population sizes have increased [6,7,8,9]. Despite the warming trend, Antarctica is still among the coldest regions on the planet [10], and Antarctic plants must continually cope with freezing temperatures, even during the growing season [11,12]. This implies that Antarctic plants should maintain their freezing tolerance even during the growing season (summer).

*Deschampsia antarctica* Desv. (Poaceae), the only monocot that has naturally colonized Antarctica, is used to dealing with occasional daily warm temperatures since leaf temperatures can reach up to 20 °C on sunny days, even when the air temperature does not exceed 4 °C [13]. Although the rise of diurnal temperatures appears to have no effect on the freezing tolerance in this plant species [14], the foreseeable increase in the minimum nocturnal temperature (nocturnal warming) could lead to cold deacclimation [14].

Cold deacclimation entails the partial or total loss of previously acquired freezing tolerance traits [15], and is not just a passive reversal of cold acclimation, but rather a genetically and functionally distinct process [16,17]. Even though various studies have analyzed the cold deacclimation profile after a temperature increase in several species (*Arabidopsis thaliana*, *Brassica napus*, *Hordeum vulgare*, *Rhododendron anthopogon*, among others [17,18,19,20]), and identified up- and down-regulated genes during this process, they have not differentiated between the effects of diurnal or nocturnal temperature increments. In addition, the cold deacclimation dynamic could change among species [21], as well as the gene expression associated with this process.

The ‘C-repeat binding factor’ (CBF/DREB1) transcription factors are key in the regulation of a group of genes important in plants’ freezing tolerance [22,23,24]. As plants should not be expressing their cryoprotective mechanisms during cold deacclimation, it is expected that the CBF genes would be down-regulated. However, even though some of the physiological and molecular mechanisms of freezing tolerance seem to be similar in the main angiosperm clades, the genes and genetic pathways can vary widely among species [25]. For example, *A. thaliana* has 4 CBF genes, but only 3 respond to low temperatures [26], while in pooids, the number of CBF genes can exceed 20, with variable numbers in different species [27]. Thus, given the complexity of this pathway in cereals and grasses, more studies are needed to understand its functioning and interaction with variable environmental factors.

The paradox between improved growth vs. cold acclimation faces *D. antarctica* to a likely differential response against diurnal and nocturnal warming, this prompts the question about what happens with the expression of freezing tolerance-related genes. We propose that nocturnal warming down-regulates the expression of freezing tolerance-related genes in *D. antarctica* plants, while the decrease in temperatures close to 0 °C during the night is essential for transient CBF and associated gene expression, allowing *D. antarctica* plants to maintain freezing tolerance, even with a daytime temperature increase. Differentially expressed genes during the day and night experimental warming were analyzed to assess the differential effect of nocturnal warming in cold deacclimation regulation.

## 2. Results

### 2.1. RNA-Seq Analysis of Nocturnal Expressed Genes in D. antarctica

RNA-seq transcriptome analysis was carried out using RNA from *D. antarctica* leaves collected 2 h after the beginning of the nocturnal period. Three biological replicates for each treatment with and without warming were sent to the Illumina Novaseq 6000 platform for deep sequencing. The raw sequence data can be found at BioProject ID PRJNA941125.

A total of 275.3 million paired-end reads were obtained after removing the low-quality sequence and adaptor sequence. The percentage of Q30 bases was above 95.99%, while the mapping rates of all 12 libraries ranged from 86.2% to 87.6%, regarding high-quality reads (Supplementary Table S1).

After error correction and redundancy removal, de novo transcriptome assembly resulted in 193,333 transcripts corresponding to 84,162 genes, with an average GC percentage of 47.65%. The median contig length was 1127 bp, while the average contig length was 1533 bp (Supplementary Table S2). Based on benchmarking universal single-copy ortholog (BUSCO) analysis, 1549 (95.9%) of the 1614 expected embryophyte genes were identified as complete after removing redundancy, and only 4.1% were fragmented or missing (Supplementary Table S3).

### 2.2. Differentially Expressed Genes (DEGs) of Warmed vs. Cold-Acclimated Plants

To analyze the differences of the *D. antarctica* transcription levels during the nocturnal period, FPKM was used to calculate the amount of differentially expressed genes. The fold change was calculated based on a comparison of the FPKM between the cold-acclimated (CA) plants and warming treatments. The genes were considered differentially expressed when the fold change of the gene expression level was at least a two-fold change and Chi-square test (*p* < 0.05) FDR < 0.01.

**Figure 1:**
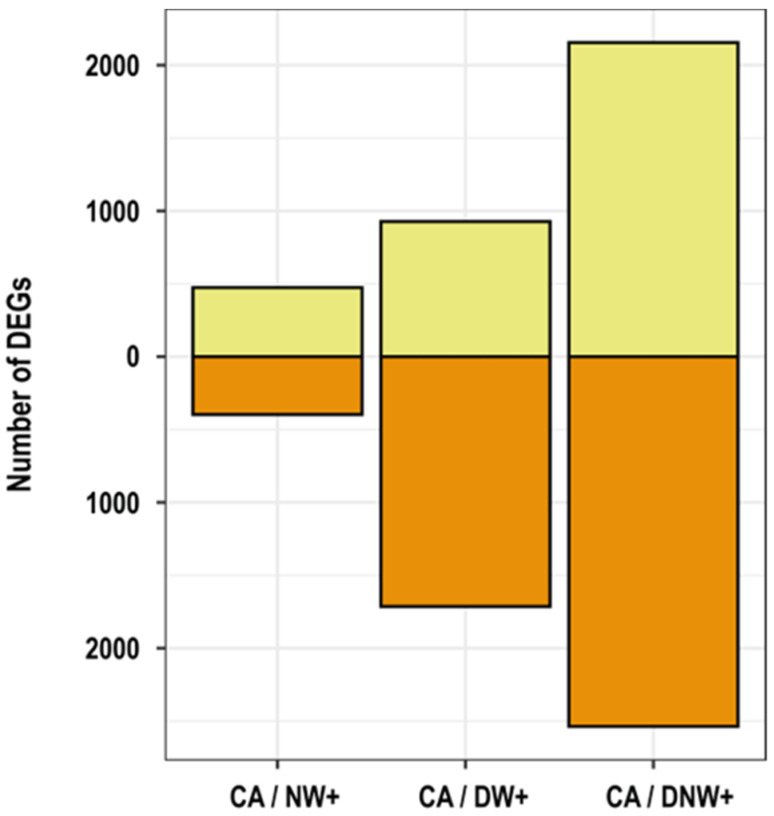
Number of differentially expressed genes (DEGs). The amounts of differentially expressed genes in warming treatments, up-regulated (light yellow) and down-regulated (orange) in relation to the control condition of cold-acclimated plants, 8 °C/0 °C (CA). Warming treatments received the following thermoperiods: 8 °C/6 °C, nocturnal warming (NW+); 14 °C/0 °C, diurnal warming (DW+); and 14 °C/6 °C, diurnal-nocturnal warming (DNW+). The plants were cultivated in chambers under controlled conditions at a photoperiod and thermoperiod of 18 h/6 h duration.

**Figure 2:**
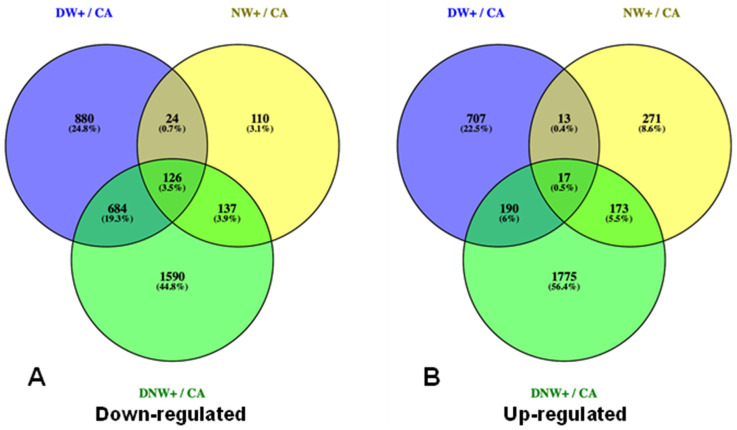
Venn diagram of the number of differentially expressed genes (DEGs). The number and percentage of down-regulated genes (**A**) and up-regulated genes (**B**) in warming treatments in relation to the control condition of cold-acclimated plants, 8 °C/0 °C (CA). Warming treatments received the following thermoperiods: 8 °C/6 °C, nocturnal warming (NW+); 14 °C/0 °C, diurnal warming (DW+); and 14 °C/6 °C, diurnal-nocturnal warming (DNW+). The plants were cultivated in chambers under controlled conditions at a photoperiod and thermoperiod of 18 h/6 h duration.

From the total of genes found in the transcriptome, only 7.77% (6537 genes) were differentially expressed 2 h after the drop in temperatures during the night period, in warmed plants. A total of 871, 4692 and 2403 DEGs were identified in plants with nocturnal warming (NW+), diurnal warming (DW+), and diurnal-nocturnal warming (DNW+), respectively, at 2 h after the temperature drop in the nocturnal period (Figure 1). Among these DEGs, 263 were down-regulated in plants with nocturnal warming, of which 52.1% was common for NW+ and DNW+ treatments only (Figure 2A), while 190 were up-regulated in plants with nocturnal warming, and 91.1% were also common for NW+ and DNW+ treatments (Figure 2B).

### 2.3. Analysis of Co-Expressed DEGs in Response to Nocturnal Temperatures

**Figure 3:**
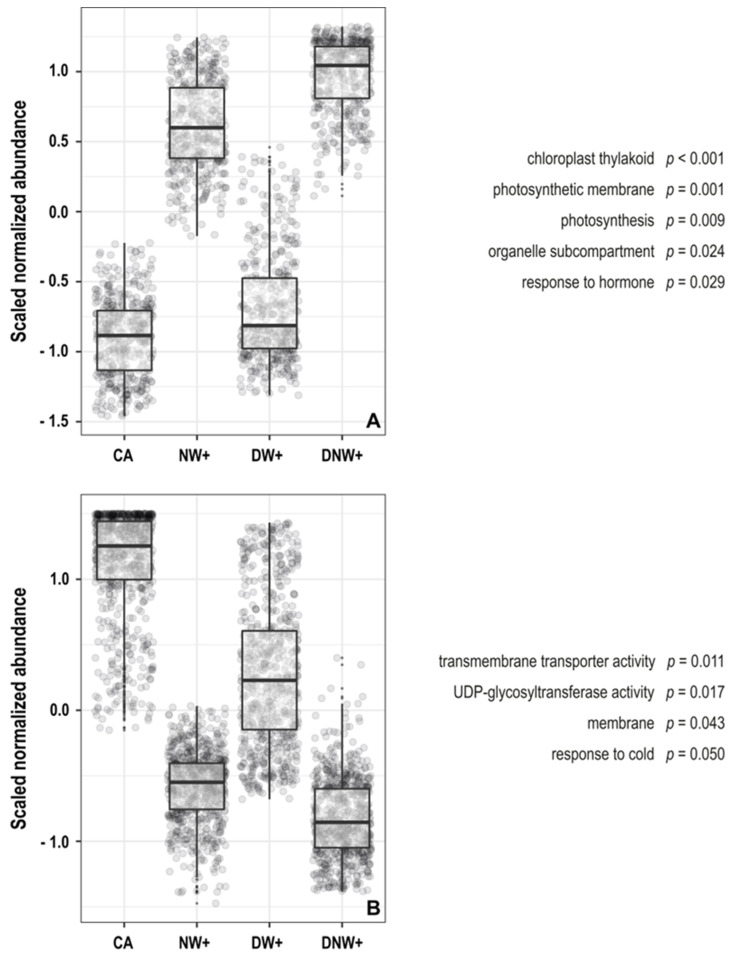
Clusters obtained from principal component analysis and self-organizing maps (PCA-SOM). The nodes group transcripts up-regulated (**A**) and down-regulated (**B**) by nocturnal warming, and the enriched gene ontology categories with their corresponding *p*-values are shown. The boxes represent the interquartile range (middle 50% of the data), its midline indicates the median value, and the whiskers indicate the ranges of the upper and lower 25%, excluding outliers. Corresponding to treatments with the following thermoperiods (day/night): 8 °C/0 °C, plants acclimated to cold (AF); 8 °C/6 °C, night heating (CN); 14 °C/0 °C, daytime warming (CD); and 14 °C/6 °C, daytime-night warming (CDN). The plants were cultivated in chambers under controlled conditions at a photoperiod and thermoperiod of 18 h/6 h duration.

The analysis of principal components (PCA) and self-organized maps (SOM), together with the grouping strategy used, allowed for the creation of eight super-nodes. Among them, two super-nodes show co-expressed genes profiles related to the presence or absence of nocturnal warming (Figure S1). The gene ontology enrichment analysis in these super-nodes showed that nocturnal-warmed plants presented a significant abundance of genes related to photosynthesis, chloroplast structures, and hormonal responses 2 h after the nocturnal period began (Figure 3A). Meanwhile, the plants without nocturnal warming presented a significant enrichment in the genes related to transporter activity and membrane structure, UDP- glycosyltransferase activity, and response to the cold (Figure 3B).

**Figure 4:**
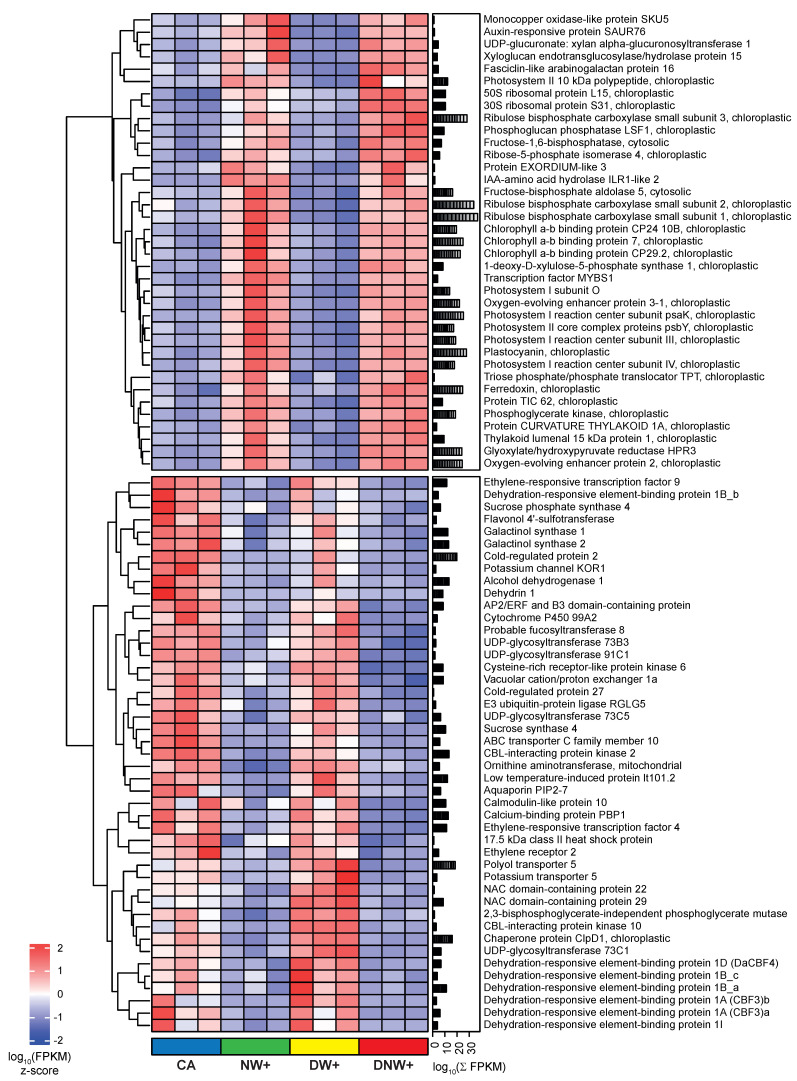
Heat-map of co-expressed genes’ response to nocturnal warming. The genes’ relative abundance is presented by the z-score of the log_10_ FPKM, after 2 h of temperature drop during the nocturnal period, of plants with a cold acclimation of 8 °C/0 °C (CA); nocturnal warming of 8 °C/6 °C (NW+); diurnal warming of 14 °C/0 °C (DW+); and diurnal-nocturnal warming of 14 °C/6 °C (DNW+). The plants were cultivated in chambers under controlled conditions at a photoperiod and thermoperiod of 18 h/6 h duration.

Likewise, the expression patterns of 1137 genes belonging to these 2 super-nodes show abiotic stress-related genes. Those related to cold tolerance are repressed in treatments with nocturnal warming. Among them, noteworthy genes include the ‘C-repeat binding factor’ (CBF or CBF/DREB1), sugar metabolism, dehydrins, and other protection-related proteins (Figure 4). Moreover, genes related to photosynthesis and vegetative growth, such as photosystems and antenna complex components, photosynthetic enzymes, and auxin regulation, among others, were induced in treatments with nocturnal warming (Figure 4).

### 2.4. Validation of RNA-Seq Gene Expression by qRT-PCR

The reliability of the transcriptome data was evaluated by quantitative real-time PCR (qRT-PCR) technology. The correlation between the log_2_ fold change of FPKM and the relative abundance determined by qRT-PCR was highly significant (R^2^ = 0.92; *p* < 0.05) for the 16 genes selected (Figure S2).

### 2.5. Nocturnal Kinetics of Interest DEGs

**Figure 5:**
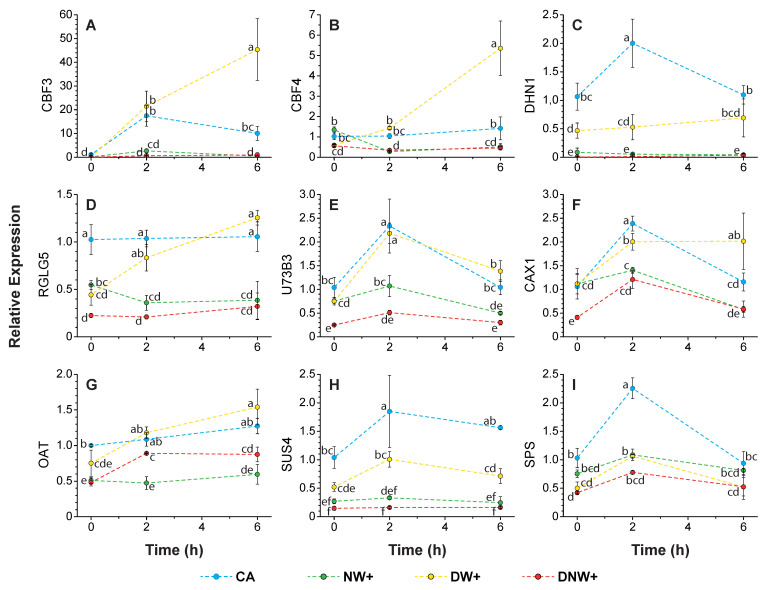
Relative expression of genes related to freezing tolerance. The variation on gene relative expression was evaluated by qRT-PCR. Values represent the fold change mean ± standard error (n = 3), in relation to cold-acclimated (CA) plants 8 °C/0 °C, at the beginning of the nocturnal period (0 h). Transcripts representing variation included: (**A**) ‘Dehydration-responsive element-binding protein 1A’ (DRE1A/CBF3), (**B**) ‘Dehydration-responsive element-binding protein 1D’ (DRE1D/CBF4), (**C**) ‘Dehydrin 1’ (DHN1), (**D**) ‘E3 ubiquitin-protein ligase RGLG5’ (RGLG5), (**E**) ‘UDP-glycosyltransferase 73B3’ (U73B3), (**F**) ‘Vacuolar cation/proton exchanger 1’ (CAX1), (**G**) ‘Ornithine aminotransferase’ (OAT), (**H**) ‘Sucrose synthase 4’ (SUS4), and (**I**) ‘Sucrose phosphate synthase 4’ (SPS4F). Warming treatments correspond to: nocturnal warming, 8 °C/6 °C (NW+); diurnal warming, 14 °C/0 °C (DW+); and diurnal-nocturnal warming, 14 °C/6 °C (DNW+), with 18 h/6 h photoperiod and thermoperiods duration. Significant differences among factors are shown as different lower cases (*p* < 0.05).

The relative gene expression during the nocturnal period was assessed with respect to the expression values average measured in CA plants, just before the nocturnal period started. The transcripts corresponding to ‘Dehydration-responsive element-binding protein 1A’ (DRE1A/CBF3), ‘Dehydration-responsive element-binding protein 1D’ (DRE1D/CBF4), ‘Dehydrin 1’ (DHN1), ‘E3 ubiquitin-protein ligase RGLG5’ (RGLG5), ‘Ornithine aminotransferase’ (OAT), ‘Sucrose phosphate synthase 4’ (SPS4F), ‘Sucrose synthase 4’ (SUS4), ‘UDP-glycosyltransferase 73B3’ (U73B3), and ‘Vacuolar cation/proton exchanger 1’ (CAX1) presented a significantly lower expression in the treatments with nocturnal warming, with respect to the CA plants, 2 h after beginning the night period (Figure 5A–I). Likewise, the aforementioned transcripts also presented significantly lower expressions in the treatments with nocturnal warming, compared to the plants with DW+, 2 h after beginning the nocturnal period, with the exception of SPS4F, (Figure 5A–I). However, in the case of SPS4F, both the CA and DW+ plants significantly increased their expression at 2 h with respect to their starting values (Figure 5I).

The expression of DRE1A/CBF3 was highly up-regulated during the night in CA and DW+ plants, but this was not modified significantly in the treatments with nocturnal warming (Figure 5A). On the other hand, the expression of DRE1D/CBF4 and RGLG5 remained stable overnight in CA plants, while their values increased in DW+ plants. Conversely, the above-mentioned genes in NW+ exhibited a reduced expression (only significantly for CBF4) to DNW+ similar values at 2 h, and remained low until the nocturnal period ended (Figure 5B,D). At the same time, DHN1 and SUS4 were the most down-regulated in the nocturnal warming treatments among the analyzed genes (Figure 5C,H).

**Figure 6:**
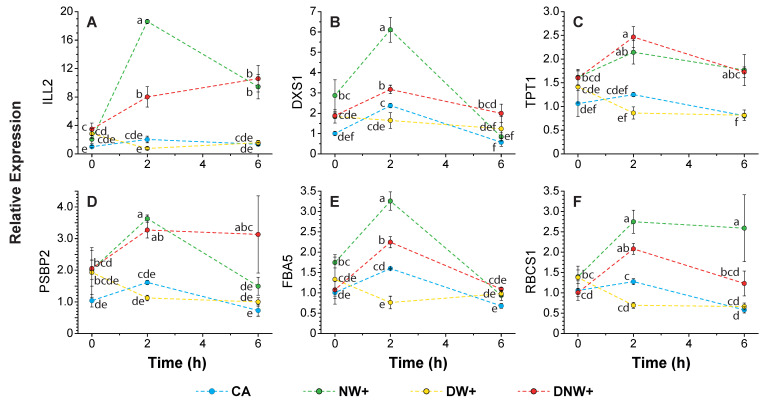
Relative expression of up-regulated genes by nocturnal warming. The variation in relative gene expression was evaluated by qRT-PCR. Values represent the fold change mean ± standard error (n = 3), in relation to cold-acclimated (CA) plants 8 °C/0 °C, at the beginning of the nocturnal period (0 h). Transcript variation shown includes: (**A**) ‘IAA-amino acid hydrolase ILR1-like 2’ (ILL2), (**B**) ‘1-deoxy-D-xylulose-5-phosphate synthase 1’ (DXS1), (**C**) ‘Triose phosphate/phosphate translocator’ (TPT), (**D**) ‘Oxygen-evolving enhancer protein 2’ (PSBP2), (**E**) ‘Fructose-bisphosphate aldolase 5’ (FAB5), and (**F**) ‘Ribulose bisphosphate carboxylase small subunit 1’ (RBCS1). Warming treatments correspond to: nocturnal warming, 8 °C/6 °C (NW+); diurnal warming, 14 °C/0 °C (DW+); and diurnal-nocturnal warming, 14 °C/6 °C (DNW+), with 18 h/6 h photoperiod and thermoperiods duration. Significant differences among factors are shown as different lower cases (*p* < 0.05).

In contrast, the transcripts corresponding to Fructose-bisphosphate aldolase 5 (FAB5), IAA-amino acid hydrolase ILR1-like 2 (ILL2), Ribulose bisphosphate carboxylase small subunit 1 (RBCS1), Oxygen-evolving enhancer protein 2 (PSBP2), Triose phosphate/phosphate translocator (TPT), and 1-deoxy-D-xylulose-5-phosphate synthase 1 (DXS1) significantly up-regulated their expression in treatments with nocturnal warming, at 2 h at the start of the nocturnal period, compared to CA and DW+ plants, (Figure 6A–F). Highlighting that, ILL2, DXS1, and FBA5 presented a significantly higher up-regulation in NW+ plants (Figure 6A,B,E). The transcripts corresponding to ILL2 and TPT1 maintained their significantly higher expression until the overnight period ended (Figure 6A,C). The ILL2 expression presented the higher increment during the night in plants with nocturnal warming (Figure 6A), with 9 time increments in NW+ plants at 2 h after the nocturnal period began, compared to its initial value.

## 3. Discussion

In the current global warming context, with more frequent drastic and stochastic temperature changes, such as heat waves and spring frosts, the early cold deacclimation of plants becomes more relevant, since leaf tissues or the whole plant may become vulnerable to subsequent freezing events, threatening their survival [21,28]. Our findings suggest that only nocturnal warming is capable of down-regulating freezing tolerant-related genes and up-regulating growth promotion and carbon assimilation-related genes, inducing cold deacclimation in *D. antarctica*.

Among the freezing tolerance-related genes that were only repressed by nocturnal warming were the CBF transcription factors, which regulate the expression of a group of genes essential in plant freezing tolerance, the CBF-regulon [22,23,24,29]. In particular, the ‘Dehydration-responsive element-binding protein 1A’ (DREB1A/CBF3) transcription factor was the most induced overnight, increasing its expression 17.6 times in CA plants 2 h into the nocturnal period, and up to 2457 times in plants with CD at the end of the night. This could suggest that DREB1A/CBF3 has a very important role in maintaining freezing tolerance in plants with nocturnal temperatures below the cold deacclimation threshold, even when CBF transcription factors show a high functional redundancy [23,24]. It also has been postulated that the expression of CBFs needs to be transient, and their transcripts return to basal levels a few hours after their induction at low temperatures [30]. In accordance with the above, the treatment with CD, which presented the highest levels of transcription for most of the CBF analyzed, also presented the highest levels of coding transcripts for the 14-3-3 protein (results not shown), which has been postulated to function as an intermediate in the CBFs’ ubiquitination modification for their subsequent degradation via the proteasome [30].

The CBF-regulon controls the expression of a diverse group of genes, and among them are other transcription factors, channel proteins and membrane transporters, enzymes related to sugar and proline metabolism, etc. [22,23,29]. Among these genes is the ‘Vacuolar cation/proton exchanger 1’ (CAX1), which has been reported to increase expression in cold-acclimated plants, such as the CA and DW+ plants in this study, and is key in the regulation of intracellular Ca^2+^ [31], one of the second messengers responsible for transmitting the cold signaling [32,33]. Some gene isoforms coding for sucrose synthase (SUS) enzymes [24,34] are also part of this regulon, and they degrade the sucrose molecule yielding UDP-glucose through a reversible reaction, allowing for its incorporation into more complex molecules synthesis, such as cellulose during the thickening of cell walls [35], or moving onto catabolic pathways as a result of an increased energy demand in the face of abiotic stress [36]. Sucrose synthase has also been found to have a predominant role in sucrose loading and unloading in phloem transport [37,38]. Both situations could be occurring in CA and DW+ plants, which would explain the induction of SUS4 in these treatments, since the cell walls’ rearrangement is an expected process in *D. antarctica* cold-acclimated plants [39], and cold acclimation is also an energetically demanding process, needing to mobilize sucrose to sinks organs.

Dehydrins gene expression are also regulated by CBF transcription factors [23,24]. This agrees with dehydrins transcript’s drastic reduction in plants with nocturnal warming, and with their protein levels reduction during the cold deacclimation of *D. antarctica* and other pooids (*Poa annua*, *Agrostis stolonifera*, and *Cynodon* spp.) [14,40,41,42,43]. Dehydrins are associated with freezing tolerance due to their ability to stabilize cell membranes, preventing the lipid bilayer transition to the hexagonal phase at freezing temperatures, as a result of the lipid interaction in a medium with less polarity, resulting leakage of electrolytes, and other essential cytoplasm components [44].

Proline accumulation has also been associated with cold acclimation and tolerance to low temperatures in *Arabidopsis thaliana*, *Chrysanthemum dichrum*, and *Lolium perenne*, among others [45,46,47,48]. In this sense, it has been described that the gene coding for ‘δ-1-pyrroline-5-carboxylate synthase 2’ (P5CS2), a key enzyme for proline synthesis, is regulated by CBFs [23]. However, the P5CS2 gene reduced its expression in all warming treatments (results not shown), while the ‘Ornithine aminotransferase’ (OAT) gene reduced its expression in plants that received nocturnal warming. The mitochondrial enzyme Ornithine aminotransferase is part of an alternative proline synthesis pathway usually activated by abiotic stress, dehydration, and oxidative stress mainly [49,50]. The OAT enzyme transforms ornithine into glutamyl-5-semi-aldehyde (GSA) for its subsequent conversion to pyrroline-5-carboxylate (P5C), which is exported to the cytoplasm and continues its conversion to proline [49]. Although the OAT genes’ expression is not significantly affected during *Chrysanthemum dichrum* cold acclimation [47], its expression was reduced in *D. antarctica* cold-deacclimated plants. This could be related to the possible existing interspecific differences, but also reaffirms the postulate that cold deacclimation is more than a passive regression from a previous cold-acclimated state [21].

Both tissue dehydration and oxidative stress can be the result of low temperature exposure, since the freezing of apoplastic fluid results in severe cell dehydration, the formation of ice crystals that cause cell membrane rupture [51], and the release of electrolytes and free radicals [52]. In this sense, the synthesis of flavonols, flavonoids, and phenylpropanoids has been proposed in *D. antarctica* as a way to increase the antioxidant capacity of cold-acclimated plants [39]. Our results confirm that ‘flavonol 4′-sulfotransferase’ and various ‘UDP-glycosyltransferase’ genes (UGT73B3, UGT73C1, UGT73C5, UGT91C1) that encode enzymes related to these antioxidant molecules synthesis pathways [53,54,55,56,57] were co-expressed together in freezing tolerant plants (CA and DW+), while their expression was down-regulated during cold deacclimation as result of nocturnal warming.

Another gene whose expression is modified by the cold is ‘E3 ubiquitin-protein ligase RGLG5’ (RGLG5), which together with RGLG1, is responsible for phosphatase 2CA (PP2CA) ubiquitination and its degradation [58], and PP2CA is a recognized repressor of the hormonal abscisic acid (ABA) synthesis pathway [58,59]. Our results show that RGLG5 transcripts were down-regulated in cold-deacclimated plants, while their expression was higher and stable in CA plants, and it gradually increased overnight in DW+ plants. This could imply a reduction in *D. antarctica* leaf tissue ABA concentration in cold-deacclimated plants by nocturnal warming, while the ABA concentration of the leaf tissue from DW+ plants could be similar to that of cold-acclimated plants at the end of the night. However, more studies are needed to fully validate this idea. In accordance with the previous approach, although the CBF-regulon expression corresponds to an ABA-independent pathway in response to the cold [60], its expression, as well the expression of other cold-regulated genes (COR), requires ABA presence to potentiate its induction by the cold [61]. This has been related to the presence in the promoter regions of the freezing tolerance genes of the CRT/DRE elements, and the sequences called ‘abscisic acid-responsive element’ (ABRE) [60].

The CBFs’ expression is not only related to the induction of freeze tolerance genes, but also to the repression of a group of genes related to plant growth [24]. *Arabidopsis thaliana* plants overexpressing one or more CBF genes exhibit constitutive freezing tolerance, slow growth, and a dwarf phenotype [62]. In addition, rice plants overexpressing the DaCBF4 gene presented a greater cold and drought tolerance than wild type [63], favoring the up-regulation of dehydrins and other LEA proteins, while down-regulating the expression of several proteins related to photosynthesis such as Chlorophyll A-B binding protein, 10 kDa chloroplast precursor polypeptide of photosystem II, 44 kDa reaction center protein of photosystem II, and the D2 protein of photosystem II [63]. Similar results were found in our study of plants that maintained freezing tolerance compared to those that were cold-deacclimated by nocturnal warming. This could suggest that DaCBF4 and other DaCBFs directly repress or promote the transcription of other repressors over a photosynthesis-related group of genes, given the functional redundancy found among CBFs genes [23,24].

Even though the transcription of genes related to photosynthetic activity does not necessarily imply a higher photosynthetic rate, it has been reported that night-warmed plants exhibited an increase in CO_2_ assimilation during the day [64,65]. Additionally, the increased expression of carbohydrate metabolism-related genes, such as the triose phosphate/phosphate translocator and Fructose-bisphosphate aldolase 5 in plants with nocturnal warming, suggests that a remobilization process of previously accumulated photo-assimilates occurs at night to obtain energy and/or to be used as intermediates in biosynthetic processes, as proposed by Turnbull et al. [64]. This nocturnal remobilization of photo-assimilates does not seem to be directed towards sucrose synthesis, since the expression of sucrose phosphate synthase transcripts did not vary throughout the night in plants with nocturnal warming. Previous results in *D. antarctica* plants under experimental laboratory conditions with nocturnal warming show a reduction in the ratio between respiration and carbon assimilation, mainly due to a greater increase in CO_2_ assimilation [65], but this increase in CO_2_ assimilation is not reflected in the accumulation of soluble carbohydrates, since there is also a reduction in the amount of sucrose accumulated [14]. All the above suggests that the accumulated carbohydrates and the recently assimilated CO_2_ were used in other biological processes, potentially including vegetative growth. This idea is consistent with the increased expression of genes related to the hormonal response in plants with nocturnal warming, such as ‘IAA-amino acid hydrolase ILR1-like 2’, which allows for the release of previously accumulated auxins in its active form, promoting plant growth [66,67].

The relationship between the consumption of accumulated soluble carbohydrates, with the activation of photosynthetic metabolism and vegetative growth, has been previously associated with the cold deacclimation process [16,21,68]. Usually, cold deacclimation occurs during the spring season, and for some species, it could be a passive process [17]. However, *D. antarctica* plants should maintain their freezing tolerance for the whole year, since they need to deal with freezing temperatures during the growing season (summer) [11,12]. Therefore, when cold deacclimation occurs in *D. antarctica* (at least 7 °C increase in LT_50_), it was promoted by nocturnal warming [14], and this should be an active process, as was also proposed by Wójcik-Jagła et al. [17] for winter barley submitted to warm periods during the winter season. This active cold deacclimation process involves a complex gene regulation that was activated by nocturnal warming only, as has been shown in this laboratory study. Even *D. antarctica* plants cold deacclimated by nocturnal warming maintain a certain freezing resistance that prevents serious damage by the Antarctic spring and summer freezing events [14]. However, it is not possible to predict the effect that the vegetative growth promotion by nocturnal warming should have on other physiological processes, such as reproduction and seed maturity. While most of the researchers found that nocturnal warming reduces the reproductive success of cereals [69], others report opposite results [70]. Moreover, *D. antarctica* plants in the field are exposed to other variables beside temperature that can influence their physiological response, such as wind intensity, variable frequency, nutrient availability, and the intensity of freezing events, among others. Therefore, this raises questions about the relative contribution of nocturnal warming to induce the cold deacclimation process of *D. antarctica* plants under field conditions.

## 4. Materials and Methods

### 4.1. Plant Material and Warming Treatments

*Deschampsia antarctica* Desv. plants were collected near the Henryk Arctowski Polish Scientific Station, King George Island, Maritime Antarctic (62°09′41″ S–58°28′10″ W) during the 2018 growing season. Three individual plants (biological replicates) were vegetatively propagated, as previously described by Bravo et al. [71], in 400 mL pots with soil mixture 3:2:1 (vegetal soil:vermiculite:peat), with 18 h/6 h of light/dark photoperiod, in a controlled climate room at 13 °C constant temperature. These plants (four clones for each biological replicate) were cold-acclimated during 28 days with a gradual temperature reduction in the 18 h/6 h day/night thermoperiod, as described by López et al. [14]. Then, groups of pots were transferred to four thermoperiods treatments that were established with the following temperature regimes: (CA) maintained at 8 °C/0 °C day/night, the control treatment where plants remain in the final cold acclimation condition; (NW+) only nocturnal warming at 8 °C/6 °C day/night; (DW+) only diurnal warming at 14 °C/0 °C day/night; and (DNW+), both diurnal and nocturnal warming at 14 °C/6 °C day/night. Each treatment contained clones of the 3 biological replicates. The maximum and minimum temperatures of the above-mentioned thermoperiods were selected based on microenvironmental data recorded in the field, near the Henryk Arctowski Polish Antarctic Station, King George Island. It has been reported that an increase of 6 °C in the minimum nocturnal temperature leads to the cold deacclimation of *D. antarctica*, unlike the same increase in the maximum daytime temperature [14]. The plants remained in these conditions for 14 days.

During the fourteenth night, the plants were kept at the maximum temperature of their corresponding thermoperiod until the end of the diurnal photoperiod. Samples were collected from 3 plants for each treatment during the night. First, just before the nocturnal period began, which was considered time 0 h, the initial state before the beginning of the night and the temperature dropped. Next, the plants were moved into dark culture chambers with their corresponding nocturnal thermoperiod of each treatment (0 °C CA and DW+; 6 °C NW+ and DNW+). After 2 h, when the expression of the CBF genes should be the maximum [26], the same plants were sampled again, and also at 6 h, just before the diurnal photoperiod began and the temperature rose, in order to analyze the effect of nocturnal warming on CBFs and freezing tolerant-related genes.

### 4.2. Total RNA Extraction and Quantification

Total RNA extraction and purification was performed using a RNeasy ^®®^ Plant Mini Kit (QIAGEN), according to the manufacturer’s recommendations. Total RNA concentration was determined using an Infinite M200 NanoQuant spectrophotometer measured at 260 nm, and the A260/A280 absorbance ratio was used to estimate its purity.

Additionally, from extracted RNA, cDNA synthesis was performed with the commercial AffinityScript qRT-PCR kit (Stratagene, Cedar Creek, TX, USA), following the manufacturer’s instructions. Reverse transcription was performed from 1 μg of total RNA, using oligos-dT and random primers as primers in a final volume of 20 μL. At the end of the reaction, the cDNA in solution was diluted in a 1:10 ratio with nuclease-free water, before being aliquoted and stored at −80 °C until use.

### 4.3. Sequencing and De Novo Transcriptome Assembly

A total of twelve samples were sequenced in a Novaseq 6000 platform, for 150 cycles in the paired-end mode at Novogene facilities. The resulting fastq files were processed using Trimmomatic v0.38 [72] to remove residual adapters and low-quality sequences. These high-quality reads were used to construct a de novo assembly of a *Deschampsia antarctica* transcriptome using Trinity v2.12 [73].

### 4.4. Transcriptome Assessment and Abundance Estimation

The raw assembly was clustered at 95% similarity using CD-HIT [74], and the completeness was evaluated using BUSCO v5 (Simao et al., 2015), which queried a collection of proteins (Embryophyta odb10) to the assembled transcripts by similarity. The relative abundance estimation was calculated using RSEM v1.2.26 [75].

### 4.5. Differential Gene Expression and Co-Expression Analysis

Changes in relative RNA abundance were analyzed with Bioconductor package DESeq2 [76] in the R statistical environment. The significance of the changes in gene expression were judged using a False Discovery Rate (FDR) of less than 0.05 and a minimum fold change (FC) of 2 as the threshold. The expression patterns in the differentially expressed genes were clustered using dimensionality reduction with Principal Component Analysis (PCA), followed by a 8 × 8 Self-Organizing Map (SOM) using one hundred training iterations, as implemented in the Kohonen package in R [77]. The resulting SOM nodes were grouped using a hierarchical clustering approach with a branch length cutoff of 2.2, resulting in 9 super-nodes. The abundance patterns of each super-node were visualized in a boxplot to associate their accumulation with any treatment.

### 4.6. Transcriptome Annotation and Enrichment Test

The resulting genes were compared by homology into the UniProt/SwissProtKB database using BLAST+ with an e-value of 1 × 10^−10^ as the threshold. The ontology assignment was performed using the PANTHER (version 2020_4) classification system [78], from EMBL. Each super-node was analyzed for the enrichment of gene ontologies with a false discovery rate (FDR) of 0.05 using GOSeq [79].

### 4.7. Quantitative Real-Time (qRT-PCR) Analysis

The expression patterns of DEGs were analyzed by qRT-PCR, performed in a Stratagene AriaMX thermocycler (Agilent, Santa Clara, CA, USA) using the previously obtained cDNA and Brilliant II SYBR Green QPCR Master mix reagent (Stratagene, Cedar Creek, TX, USA), according to the manufacturer’s instructions. The gene-specific primers (Table S4) used for the qRT-PCR analysis were designed using the Primer-BLAST tool (http://www.ncbi.nlm.nih.gov/tools/primer-blast accessed on 12 October 2022). The genes Polyadenylate-binding protein2 (PAB2) and Eukaryotic translation initiation factor (IF4E4) were used as internal references to normalize the expression data. The relative expression levels were calculated according to the method proposed by Pfaffl [80], based on the gene primer efficiency of the e^−ΔΔCT^ (cycle threshold). To validate the repeatability of the RNA-seq data, 16 DEGs were selected for verification by qRT-PCR, using the cDNA obtained from the same sample that was sent to the sequence. The relative expression was estimated with respect to their values in the control treatment (CA), 2 h into the nocturnal period. In contrast, the kinetic variation of genes expression during the night was evaluated with respect to their values in the control treatment (CA) at 0 h just before the nocturnal temperature began.

## 5. Conclusions

The evidence obtained in the present study confirms that nocturnal warming is key in the cold deacclimation process through the down-regulation of freezing tolerance-related genes, and the up-regulation of photosynthetic and growth-related genes in *D. antarctica*. Furthermore, the expression of the CBF transcription factor and its regulon during the night is necessary to maintain the previously acquired freezing tolerance. However, more studies are necessary to understand the consequences that regional warming will have on *D. antarctica* field populations. In this sense, it would be interesting to evaluate how gene expression is affected by nocturnal warming under field experimental conditions.

## Data Availability

The sequencing data was deposited in the National Center for Biotechnology Information (NCBI) and can be accessed via BioProject ID PRJNA941125.

## Supplementary Materials

The following supporting information can be downloaded at: https://www.mdpi.com/article/10.3390/ijms241311211/s1.
