# Supplementary material for: Transcriptome Analysis of Diurnal and Nocturnal-Warmed Plants, the Molecular Mechanism Underlying Cold Deacclimation Response in Deschampsia antarctica
Source: Int J Mol Sci. 2023 Jul 7;24(13):11211. doi: 10.3390/ijms241311211 (PMC10342576; doi:10.3390/ijms241311211)
Supplement: Supplementary file 1 [file ijms-24-11211-s001.zip › Supplementary Figures.pdf]

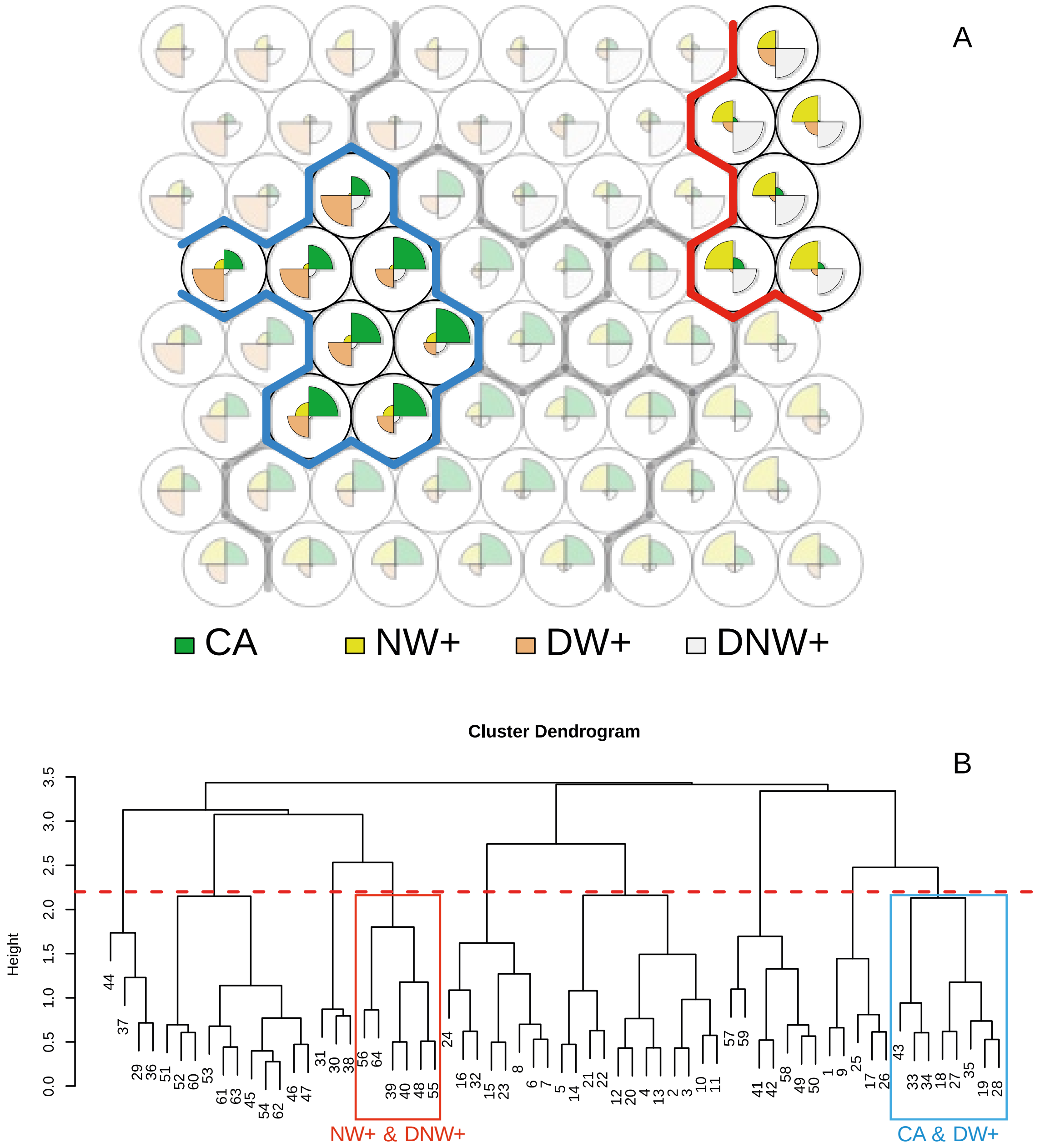

Figure S1. Super-nodes clustering. Visual representation of gene co-expression in the 64 original nodes and their clustering in the 8 super-nodes (A). Clustering dendrogram of the 64 original nodes, demarcating the cut line at height 2.2 with a red dashed line (B). The super-node grouping up-regulated genes by nocturnal warming is outlined in red, meanwhile the super-node grouping down-regulated genes are outlined in blue. The treatments were: 8°C/0°C, plants cold acclimated (CA); 8°C/6°C, nocturnal warming (NW+); 14°C/0°C, diurnal warming (DW+) and 14°C/6°C, diurnal-nocturnal warming (DNW+). The plants were cultivated in chambers under controlled conditions at a photoperiod and thermoperiod of 18h/6h duration.

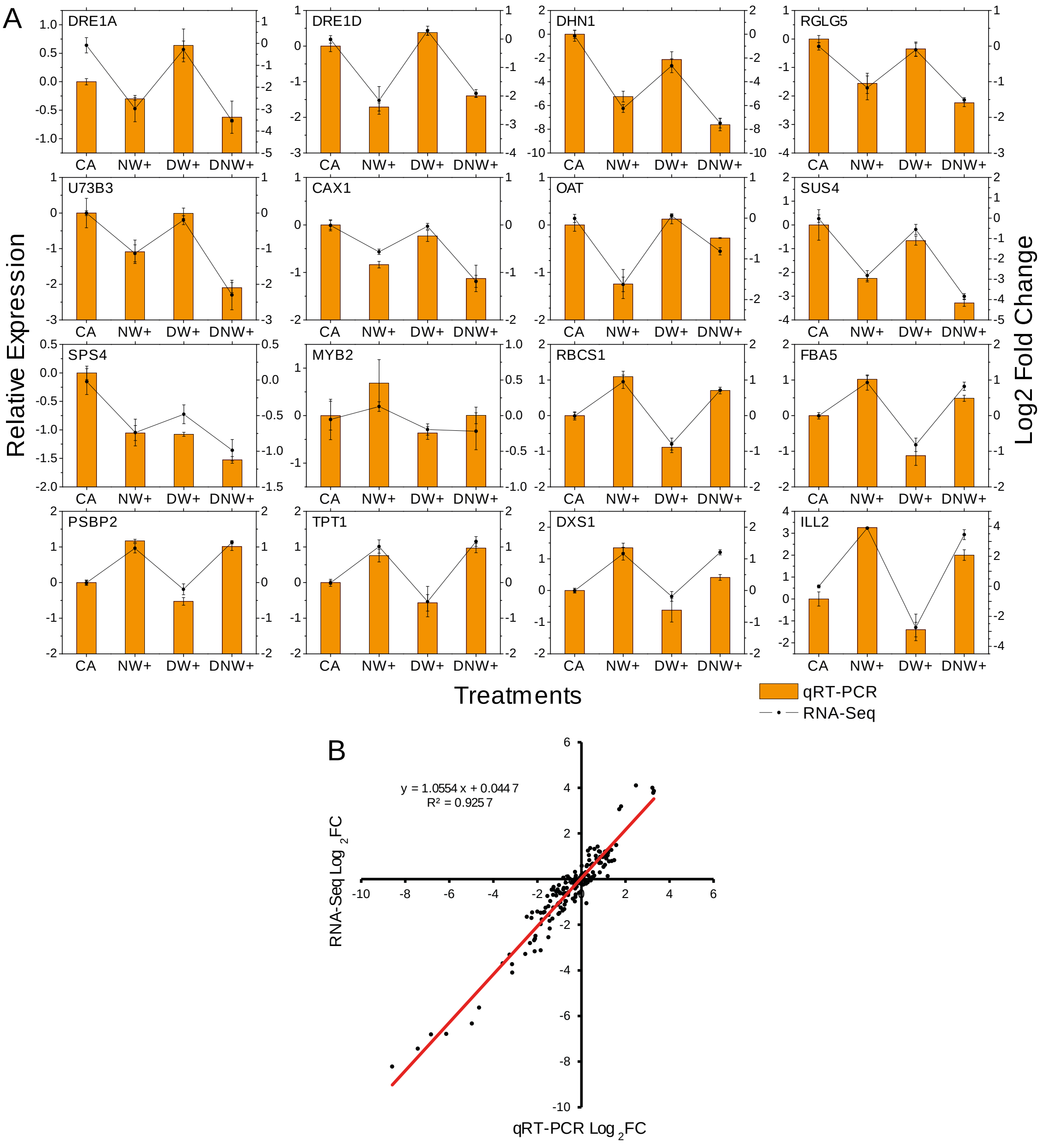

Figure S2. Transcriptome validation. Relation between the Log_2 Fold change (FC) of gene expression evaluated by real-time PCR (qRT-PCR) and Log_2 FC of FPKM (A). Bars and circles show the mean ± standard error (n = 3) for qRT-PCR and RNA-seq respectively, corresponding to experimental treatments with the following day/night thermoperiod: 8°C/0°C cold-acclimated (CA); 8°C/6°C nocturnal warming (NW+); 14°C/0°C diurnal warming (DW+) and 14°C/6°C diurnal and nocturnal warming (DNW+), with 18h/6h photoperiod and thermoperiods duration. Significant Pearson correlation ($p < 0.05$) between qRT-PCR and RNA-seq of 16 differentially expressed genes (B).
