# Supplementary material for: Transcriptome Analysis of Diurnal and Nocturnal-Warmed Plants, the Molecular Mechanism Underlying Cold Deacclimation Response in Deschampsia antarctica
Source: Int J Mol Sci. 2023 Jul 7;24(13):11211. doi: 10.3390/ijms241311211 (PMC10342576; doi:10.3390/ijms241311211)
Supplement: Supplementary file 1 [file ijms-24-11211-s001.zip › Supplementary Tables.pdf]

Supplementary Table S1. Total number of raw reads, high-quality reads per sample, and their mapping rate within *Deschampsia antarctica* transcriptome

Sample	*raw reads*	*High quality reads*	*Mapped reads*	*Mapping rate (%)*
E1	20,456,461	19,637,272	16,946,758	86.30
E2	20,911,828	20,089,989	17,323,597	86.23
E3	24,332,760	23,472,782	20,437,217	87.07
F1	24,659,473	23,781,109	20,747,380	87.24
F2	24,947,240	24,390,972	21,318,883	87.40
F3	23,132,517	22,642,675	19,845,043	87.64
G1	24,107,540	23,523,818	20,374,801	86.61
G2	23,367,878	22,862,486	19,854,336	86.84
G3	24,445,058	23,813,002	20,645,369	86.70
H1	30,212,625	29,515,612	25,920,826	87.82
H2	19,821,101	19,300,281	16,630,192	86.17
H3	22,739,157	22,268,592	19,435,836	87.28

Supplementary Table S2. *De novo* transcriptome assembly of *Deschampsia antarctica* by RNA-Seq

Metric	*D. antartica transcriptome*
Trinity transcripts	193,333
**Trinity "genes"**	**84,162**
GC (%)	47.65
Contig N50 (bp)	2,255
Median contig length (bp)	1,127
Average contig (bp)	1,533
Total assembled bases	296,554,700

Supplementary Table S3. Assessment of completeness of the *Deschampsia antarctica de novo* assembly using BUSCO[+].

Summary BUSCO statistics		
**Complete BUSCOs**	**1,549**	**95.9%**
Complete and single-copy BUSCOs	288	17.8%
Complete and duplicate BUSCOs	1,261	78.1%
Fragmented BUSCOs	31	1.9%
Missing BUSCOs	34	2.2%
Total BUSCO genes *	1,614	100.0%

*Database: embriophyte_odb10

[+]BUSCO: Benchmarking Universal Single-Copy Orthologs

Supplementary Table S4. List of primers used for qRT-PCR analysis.

Gene	*Transcript name*	*Primers*		*Amplicon*
		Type	*Sequence 5′-3′*	*(pb)*
**PAB2**	*Polyadenylate-binding protein2*	FW	TGGGAGCAACACCAGGTCTC	160
		RW	CCTCCAGTTGCTCCTCGCAT	
**IF4E4**	*Eukaryotic translation initiation factor4*	FW	CACCTCGCTCAAGAAGGGCT	159
		RW	CCATTTGCGCACTCTGGGTC	
**MYB2**	*Transcription factor MYB2*	FW	ACCTGAGACGGCAGCTCTTG	184
		RW	CACGAGAGGGACTCGAGCAG	
**DRE1A CBF3**	*Dehydration-responsive element-binding protein 1A*	FW	GGTCCGCGTAGTACGATCCC	106
		RW	GACGGTTTGTTCGAGGTGCC	
**DRE1D CBF4**	*Dehydration-responsive element-binding protein 1D*	FW	GACAACGTCCTTCACAGCCG	160
		RW	GTGCCTCAACTTCGCCGACT	
**DHN1**	*Dehydrin 1*	FW	TCCAGCCGATGAAGGACGAC	175
		RW	GTCTGCTGTTGCTGCTGCTC	
**SPSA4**	*Sucrose phosphate synthase A4*	FW	TCCGGTGAACCTGAGCCAAC	138
		RW	GCGTGGTGTCAGCTGCTATG	
**SUS4**	*Sucrose synthase 4*	FW	ACGTACAGCGTCCACAGAGA	173
		RW	TGTCCAGCGAGTGGTGAACT	
**RGLG5**	*E3 ubiquitin-protein ligase 5*	FW	CGGAGCTTGCACGCAATAGG	122
		RW	GCATCGCCGAATCCAAAGCA	
**CAX1**	*Vacuolar cation/proton exchanger 1*	FW	CAGAAGAGGGAGGTGCCGAG	96
		RW	CGCGCTCATGGAGGGTAAGA	

**U73B3**	*UDP-glycosyltransferase 73B3*	FW	CGCATTCATCGAGCGGTACG	118
		RW	GACGGCTGCGTTATTGCCTC	
**OAT**	*Ornithine aminotransferase*	FW	GGTAAGTGCAGTTCTCGCGG	109
		RW	TGATGCAACTGCCACAGCAC	
**DXS1**	*1-deoxy-D-xylulose-5-phosphate synthase 1*	FW	AAGATGACGTCGGAGCGGAG	163
		RW	GGTCCTGCATTTCTGCGTCG	
**PSBP2**	*Oxygen-evolving enhancer protein 2*	FW	GTGGTGGACGGGAAGCAGTA	165
		RW	CTTCTTCGCGCCCTTGAACC	
**ILL2**	*IAA-amino acid hydrolase ILR1-like 2*	FW	CGTAGCGACGGTCTCGAAGT	177
		RW	TGCTTCTCGAACGAGGGCTT	
**RBCS1**	*Ribulose bisphosphate carboxylase small subunit 1*	FW	TTACTTGCCACCGCTCACCA	109
		RW	ACGGAACACAAACCCGACCT	
**FBA5**	*Fructose-bisphosphate aldolase 5*	FW	CACCCTCCTCAAGCCCAACA	182
		RW	TCATGGCGTCCAGGTTCTCC	
**TPT**	*Triose phosphate/phosphate translocator*	FW	ACGCTCCTGAAGCTGCTCTT	143
		RW	TGGGTAGCCGCTGCATTGAA	
